# Effect of Ultra-Micronized Palmitoylethanolamide and Luteolin on Olfaction and Memory in Patients with Long COVID: Results of a Longitudinal Study

**DOI:** 10.3390/cells11162552

**Published:** 2022-08-17

**Authors:** Pietro De Luca, Angelo Camaioni, Pasquale Marra, Giovanni Salzano, Giovanni Carriere, Luca Ricciardi, Resi Pucci, Nicola Montemurro, Michael J. Brenner, Arianna Di Stadio

**Affiliations:** 1 Department of Medicine, Surgery and Dentistry, University of Salerno, Baronissi, 84081 Salerno, Italy; 2 Otolaryngology Department, San Giovanni-Addolorata Hospital, 00184 Rome, Italy; 3 ENT and Maxillofacial Surgery Unit, Istituto Tumori G. Pascale of Naples, 80131 Naples, Italy; 4 Otolaryngology, Ars Medica, 00100 Rome, Italy; 5 Division of Neurosurgery, Sant’Andrea Hospital, Department of Neuroscience, Mental Health and Sense Organs (NESMOS), Sapienza University of Rome, 00189 Rome, Italy; 6 Oral and Maxillofacial, San Camillo Hospital, 00152 Rome, Italy; 7 Department of Oral and Maxillofacial Science, Sapienza University of Rome, 00185 Rome, Italy; 8 Department of Neurosurgery, Pisana University Hospital, University of Pisa, 56124 Pisa, Italy; 9 Department of Otolaryngology-Head and Neck Surgery, University of Michigan Medical School, Ann Arbor, MI 48109, USA; 10 Department of GF Ingrassia, Via di Santa Sofia 87, University of Catania, 95123 Catania, Italy; 11 Neuroinflammation Lab, UCL Queen Square Neurology, London WC1N 1PJ, UK

**Keywords:** anosmia, hyposmia, parosmia, palmitoylethanolamide, lutein, COVID-19, mental clouding, brain fog, memory, olfactory training, smell disorders, qualitative smell disorders, PEA-LUT, olfactory threshold, long-haul COVID syndrome, post-acute sequelae of SARS-CoV-2 infection (PASC), neuroinflammation

## Abstract

In this study, we investigated whether treatment with palmitoylethanolamide and luteolin (PEA-LUT) leads to improvement in the quantitative or qualitative measures of olfactory dysfunction or relief from mental clouding in patients affected by long COVID. Patients with long COVID olfactory dysfunction were allocated to different groups based on the presence (“previously treated”) or absence (“naïve”) of prior exposure to olfactory training. Patients were then randomized to receive PEA-LUT alone or in combination with olfactory training. Olfactory function and memory were assessed at monthly intervals using self-report measures and quantitative thresholds. A total of 69 patients (43 women, 26 men) with an age average of 40.6 + 10.5 were recruited. PEA-LUT therapy was associated with a significant improvement in validated odor identification scores at the baseline versus each subsequent month; assessment at 3 months showed an average improvement of 10.7 + 2.6, CI 95%: 6–14 (*p* < 0.0001). The overall prevalence of parosmia was 79.7% (55 patients), with a significant improvement from the baseline to 3 months (*p* < 0.0001), namely in 31 patients from the Naïve 1 group (72%), 15 from the Naïve 2 group (93.7%), and 9 from the remaining group (90%). Overall, mental clouding was detected in 37.7% (26 subjects) of the cases, with a reduction in severity from the baseline to three months (*p* = 0.02), namely in 15 patients from the Naïve 1 group (34.8%), 7 from the Naïve 2 group (43.7%), and 4 from the remaining group (40%). Conclusions. In patients with long COVID and chronic olfactory loss, a regimen including oral PEA-LUT and olfactory training ameliorated olfactory dysfunction and memory. Further investigations are necessary to discern biomarkers, mechanisms, and long-term outcomes.

## 1. Introduction

Severe acute respiratory syndrome coronavirus 2 (SARS-CoV-2) is estimated to have infected over a half billion people worldwide, and the post-acute sequelae of SARS-CoV-2 infection (PASC), or long-haul COVID syndrome (“long COVID”), is an impending public health crisis. Long COVID encompasses a constellation of persistent symptoms, often attributed to neuroinflammation, that persist for at least three months after the resolution of the acute symptoms of COVID-19 [1]. Among the more common symptoms are fatigue, mental clouding (“brain fog”), olfactory or gustatory dysfunction, and shortness of breath or cough. Our recent double-blinded clinical trial showed that more than 25% of COVID-19 long-haulers can suffer from disrupted smell function (hyposmia, anosmia, or parosmia/cacosmia) [2].

Difficulty with memory, concentration, decision making, and subjective cognitive impairment or behavioral changes (commonly referred to as “brain fog”) are reported by almost a third of COVID-19 patients [3,4]. These cognitive impairments have broad effects on daily functioning and are common in non-hospitalized patients with mild COVID-19 [3,4]. Although the pathogenesis of brain fog is not fully understood, it has been linked to neuroinflammation associated with the spread of SARS-CoV-2 to the higher brain centers. Brain fog is thought to primarily arise from the diffusion of inflammation within the olfactory bulbs to other brain regions or, less commonly, from the direct infection of parenchymal brain tissue; systemic inflammation induced by SARS-CoV-2 might also have a role [5,6,7,8].

The interrelationship of olfactory dysfunction with other long COVID symptoms remains poorly understood, and few evidence-based therapies are available. We previously reported that mental clouding and headache were associated with more severe olfactory loss in adult patients with post-COVID olfactory dysfunction. This finding, which is consistent with the neuroinflammatory hypothesis of long COVID symptoms [9], provides a rationale for targeting neuroinflammation to promote recovery. Palmitoylethanolamide and luteolin (PEA-LUT) have anti-inflammatory and neuroprotective properties [10,11,12]. In the present study, we investigated whether treatment with PEA-LUT, with or without olfactory training, was associated with an improvement in measures of olfactory function and mental clouding among patients affected by long COVID.

## 2. Materials and Methods

### 2.1. Study Population and Demographic Data

This longitudinal study was conducted in a tertiary referral hospital in Rome from April 2021 to October 2021. To recruit patients, we used word-of-mouth communication among clinicians and calls through newspapers, television, and the internet (mass media). All centers used the same procedures and protocols. Patients were assigned a number at the time of recruitment, and they were informed that the purpose of the study was to investigate approaches for treating persistent loss of smell after COVID-19. Patients were also advised that, after the baseline assessment, they would have a follow-up olfactory assessment at 90 days, with up to two possible intermediary olfactory assessments, as dictated by protocol. Patients were instructed that their participation was voluntary and that they could withdraw from the study at any time. A single physician at each center performed an endoscopy to rule out baseline pathologies (e.g., polyps or tumors), and a second physician performed olfactory testing using validated measures of threshold, discrimination, and identification scores. Self-reported data on mental clouding/brain fog were also collected. The physician who performed the nasal endoscopy had knowledge of the experimental groups and did not participate in the olfactory assessment. All the collected data were anonymized and recorded on a protected Excel sheet shared by all the centers (Google (Mountain View, CA, USA)). Study participants were included or excluded based on the following criteria:

#### 2.1.1. Inclusion Criteria

Eligible patients for the study included outpatients, ages 18 to 80 years, with a confirmed history of COVID-19 (positive nasopharyngeal swab for SARS-CoV-2), and anosmia/hyposmia confirmed with the 16-pen version of the Burghart Sniffin’ Sticks psychophysical test (I score 0–16), with olfactory impairment persisting ≥ 180 days (6 months) after a subsequent negative COVID-19 nasopharyngeal swab. Chronic olfactory dysfunction after SARS-CoV-2 thus served as a marker for long COVID, and signed informed consent was obtained from all the individuals opting to participate in the study.

#### 2.1.2. Exclusion Criteria

Exclusion criteria included a previous history of olfactory–gustatory disorders, previous known/perceived/referred disorders of memory, active chemotherapy or treatment with estrogen inhibitors (aromatase), impaired cognitive function, history of neurodegenerative disease (Alzheimer’s and Parkinson’s disease), medical therapy with known detrimental effects on olfactory function, the presence of active rhinologic disorders (sinusitis, rhinosinusitis, sinonasal polyposis, atrophic rhinitis, allergy) at the time of enrollment, history or chemoradiotherapy of the head and neck region, history of stroke or neurotrauma, severe nasal blockage from stenosis or deformity, severe psychiatric illness (e.g., schizophrenia, bipolar disorder, olfactory hallucination), previous sinonasal or nasopharyngeal tumors, or corticosteroid therapy used to treat olfactory dysfunction within the previous 30 days. Additionally, any of the patients who were using medications with anti-inflammatory or immune-modulating effects that could either independently reduce inflammation or interfere with PEA-LUT were excluded from the study.

### 2.2. Demographic Data Extraction

For each patient, the following demographic data were collected: sex, age, major disease, tobacco/alcohol use, medications, prior treatment for an olfactory disorder, and the time elapsed since the negative COVID-19 test. A medical record was created for each patient that included the patient’s general medical and family health history, details on COVID-19 illness (date and symptoms at the onset, date of positive and negative PCR testing, treatments used during the infection, persistent symptoms, treatments used after COVID-19 resolution), and any history of COVID-19 vaccination. To this medical history record, we added a section for recording detailed data on the identification of smell alterations. We verbally explained the differences between anosmia, hyposmia, parosmia, and phantosmia to patients. Patients were then queried about their own history. At the end of the study (three months after randomization and the initiation of therapy), the data were extracted and analyzed by a statistician, following the procedures stipulated by the study coordinator (A.D.S.).

### 2.3. Experimental Groups

The 69 consecutive patients enrolled in the study were assigned to 3 groups, as follows:

- Individuals previously exposed to olfactory training

Previously trained group (PEA-LUT plus olfactory training): All the individuals with prior olfactory training received a daily PEA-LUT oral supplement and continued olfactory training. The supplement contained co-ultra-micronized PEA 700 mg and luteolin 70 mg (Glialia^®^, Epitech Group SpA, Saccolongo (PD), Italy) administered as a single sublingual dose, 5–10 min before breakfast. Olfactory training entailed stimulation using four 100% organic essences (lemon, rose, eucalyptus, and cloves) administered three times per day for 6 min each session; stimulation consisted of smelling an odor for 5–8 s, then 40 s of relaxation, and then, new stimulation for 4–6 s with another essence. This short duration was used to avoid the “saturation” of the olfactory receptors [2]. Subjects performed this regimen for 90 consecutive days.

- Individuals not previously exposed to olfactory training (Training-Naïve)

These individuals were allocated to PEA-LUT with or without olfactory training:

Training-Naïve 1: (PEA-LUT plus olfactory training): patients consumed one sublingual sachet of PEA-LUT (700 mg + 70 mg) per day and performed olfactory training three times a day;

Training-Naïve 2: (PEA-LUT alone): patients consumed one sublingual sachet of PEA-LUT (700 mg + 70 mg) per day and underwent no additional intervention.

### 2.4. Nasal Endoscopy Assessment of Olfactory Dysfunction

All the patients underwent nasal endoscopy to exclude nasal conditions that might confound or otherwise interfere with olfactory testing or treatment interventions. If masses, sinonasal polyposis, active infection, or other rhinologic disorders were identified on nasal endoscopy, the patient would be excluded from the study. Patients were also queried regarding their history of prior impaired smell, history of nasal/ nasopharyngeal malignancy, history of radiation, or other anatomical abnormalities that would interfere with their sense of smell or potential influence response to therapy.

To evaluate olfactory functions, the Burghart Sniffin’ Sticks identification test (16-pen test) was performed, as directed by the manufacturer (MediSense, Sense Trading BV, Groningen, The Netherlands). The analyzed time points include T0 (baseline), T1 (1 month), T2 (2 months), and T3 (3 months). The patients’ olfactory status was classified to have normosmia (score 13–16), hyposmia (score 8–12), or anosmia (score 0–7). Additionally, patients were queried regarding any perception of altered olfaction (parosmia) or aversive smell, such as cacosmia, gasoline-type smell, or otherwise. No validated instruments are available for parosmia, so we adapted a previously published questionnaire developed for hyperosmia [13]. The questionnaire contained 52 odors, and patients were asked to score how they perceived the various odors, using a scale from 0 (normal perception) to 10 (extremely distorted smell).

### 2.5. Assessment of Memory Dysfunction

Cognitive function was assessed using the Mini-Mental State Examination (MMSE) test. To test for mental clouding, commonly called “brain fog”, we used a previously published assessment [14], in which we asked the following questions:

- Do you forget information that you recently learned, resulting in difficulty performing a task?
- Do you have to ask for information to be repeated or have an increased need for reminder notes?
- Have you had trouble in remembering common names of objects or persons?
- Has your concentration, memory, or overall mental ability deteriorated?

Three answers were possible: (a) I suffered this problem temporarily, but it resolved (no residual memory/cognitive impairment); (b) I am suffering from this problem currently (memory/cognitive alteration is still persistent); and (c) I have never experienced this problem (absence of cognitive impairment related to COVID-19). Patients were classified as suffering from mental clouding if they answered, “I am still suffering from this problem currently” for all four questions.

If they were confirmed to be suffering from brain fog, we asked them to score (self-evaluation), using a scale from 0 (none) to 10 (incapable of performing normal activity), the severity of their brain fog on the execution of their normal everyday activity.

### 2.6. Study Outcomes

The primary outcome was a change in odor identification scores over the study period. The change in odor identification scores for any individual was reported as positive if olfaction improved over the 90-day study period, negative if olfaction worsened, and zero if there was no change. Secondary outcomes were (a) a change in the self-reported parosmia scores; (b) a change in memory scores; and (c) a change in mental clouding scores. In addition, the study included provisions for the detection of adverse events in patients; clinicians conducting the study were instructed to alert the principal investigators to the patients’ intolerance of the regimen, gastrointestinal symptoms, excessive drowsiness, heart palpitations, or other symptoms.

### 2.7. Statistical Analysis

A one-way ANOVA test was used to compare odor identification scores at T0, T1, T2, and T3 across all the patients; an ad hoc Tukey test was used to compare the observation points. A two-tailed τ-test was used to compare the parosmia scores at the baseline (T0) and 3 months after treatment (T3). The same test was used to compare the mental clouding scores at the baseline (T0) and three months after treatment. Then, we analyzed the differences in n scores from T0 to T3 across groups. A two-tailed τ-test was used to compare the parosmia scores at the baseline (T0) and 3 months after treatment (T3) in each group. A one-way ANOVA test was used to compare the olfactory, parosmia, and memory scores of the three groups. Moreover, because parosmia and mental clouding did not affect all patients, a chi-squared (χ) test was performed to evaluate the differences between the three groups. Cohen’s test was performed for evaluating the effect of different sample sizes in the presence of statistically significant differences. Statistical significance was set at *p* < 0.05. All statistics were performed using Stata^®^.

## 3. Results

### 3.1. Overall

**Figure 1:**
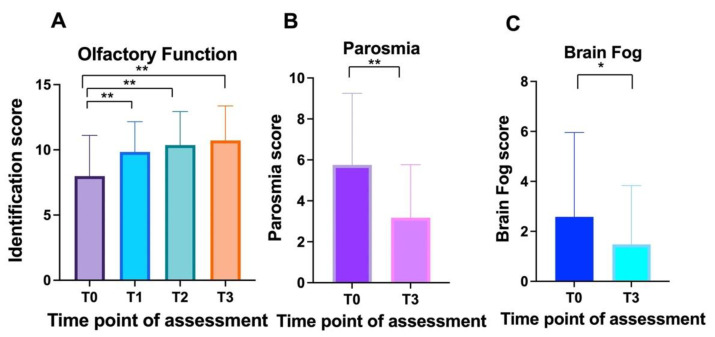
Overall trends in olfactory function, parosmia, and mental clouding in patients receiving PEA-LUT and olfactory training for post-acute sequelae of SARS-CoV-2 infection (“long COVID”): (**A**) smell identification scores at baseline (T0), T1 (1 month), T2 (2 months), and T3 (3 months) for individuals receiving therapy; (**B**) prevalence of parosmia in the cohort before (T0) and after three months of treatment (T3); (**C**) prevalence of mental clouding before (T0) and after three months of treatment (T3). **, *p* < 0.01; *, *p* < 0.05.

A total of 69 patients (43 women and 26 men) with an average age of 40.6 + 10.5 were recruited. The PEA-LUT regimen was associated with a statistically significant improvement in validated odor identification scores (ANOVA: *p* < 0.0001) (Figure 1A). Statistically significant differences in olfactory scores were observed between T0 (average 7.9 ± 3.1; CI 95%:0–12) and T1(average 9.8 ± 2.3; CI 95%: 5–13) (Tukey: *p* < 0.0001), T0 and T2 (average 10.4 ± 2.6; CI 95%: 4–14) (Tukey: *p* < 0.0001), and T0 and T3 (average 10.7 ± 2.6; CI 95%: 6–14) (Tukey: *p* < 0.0001). No statistically significant differences were observed when comparing T1 and T2 (*p* = 0.6), T1 and T3 (*p* = 0.2), and T2 and T3 (*p* = 0.8).

**Figure 2:**
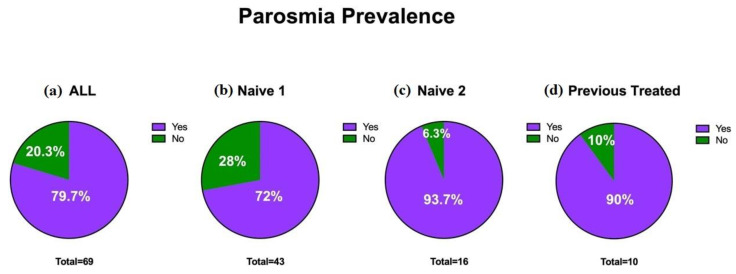
Prevalence of parosmia (T0): (**a**) overall prevalence of parosmia in the sample; (**b**) prevalence in individuals naïve to therapy 1; (**c**) prevalence in individuals naïve to therapy 2; (**d**) prevalence in previously treated individuals.

Within the cohort, 79.7% (55) patients reported parosmia (Figure 2); the odor most often identified as distorted was onion (87.2%, 48 patients), followed by fish (18%, 10 patients) and meat (14.5%, 8 people). Overall, 69% (38) patients reported distorted perceptions of either onion and fish or onion and meat. Statistically significant differences were observed in the quality of smell alteration (parosmia) between the baseline (average 5.7 ± 3.5; CI 95%:0–10) and three months after treatment (average 3.2 ± 2.6; CI 95%:0–10) (τ: *p* < 0.0001) (Figure 1B).

**Figure 3:**
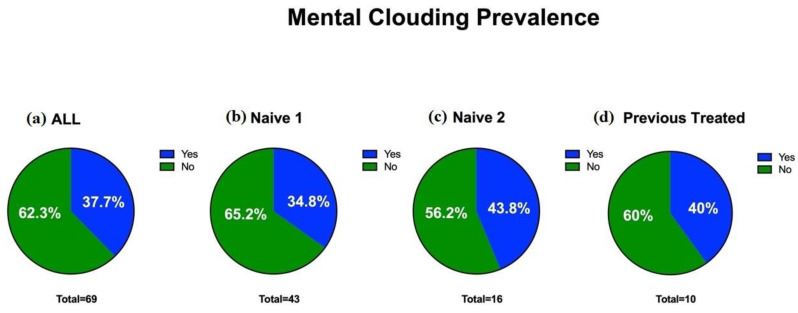
Prevalence of mental clouding in the cohort at baseline: (**a**) overall prevalence of parosmia in the sample; (**b**) prevalence in individuals naïve to therapy 1; (**c**) prevalence in individuals naïve to therapy 2; (**d**) prevalence in previously treated individuals.

Mental clouding was detected in 37.7% of the cases (26 patients) (Figure 3). Mental clouding showed a statistical reduction in severity between the baseline (average 2.6 ± 3.4; CI 95%:0–10) and T3, three months after treatment (average 1.5 ± 2.3; CI 95%:0–10) (τ: *p* = 0.02) (Figure 1C).

### 3.2. Within-Group Comparisons

**Figure 4:**
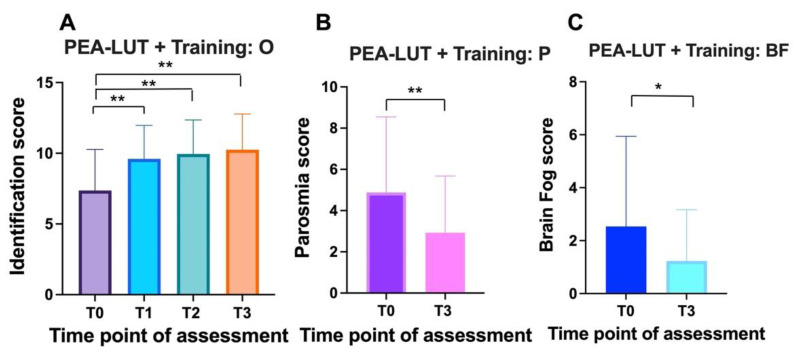
Effect of PEA-LUT treatment plus olfactory training on olfaction, parosmia, and mental clouding in patients without prior olfactory training (Naïve 1 group): (**A**) O (olfaction) results of identification test at T0 (baseline), T1 (1 month), T2 (2 months) and T3 (3 months); (**B**) P (parosmia) before (T0) and after three months (T3) of treatment; (**C**) BF (brain fog/mental clouding) before (T0) and after three months (T3) of treatment. **: *p* < 0.01; *: *p* < 0.05.

The Naïve 1 group (no prior history of olfactory training; treated with PEA-LUT plus olfactory training) comprised 43 subjects (29 women and 14 men) with an average age of 40.2 ± 10.9. These patients were affected by smell alteration for 8.5 ± 1 months on average. We observed a significant improvement in TDI scores (ANOVA: *p* < 0.0001). Statistically significant differences in TDI scores were observed between T0 (average 7.3 ± 2.9; CI 95%: 0–11) and T1 (average 9 ± 2.3; CI 95%: 5–14) (Tukey: *p* < 0.0001), T0 and T2 (average 10 + 2.4; CI 95%: 7–14) (Tukey: *p* < 0.0001), and T0 and T3 (average 10.2 ± 2.6; CI 95%: 5–14) (Tukey: *p* < 0.0001). No statistically significant differences were observed when comparing T1 and T2 (*p* = 1), T1 and T3 (*p* = 0.6), and T2 and T3 (*p* = 0.6) (Figure 4A).

Within this group, 72% (31 patients) suffered from parosmia. Statistically significant differences were observed in the quality of smell alteration (parosmia) between the baseline (average 4.9 ± 3.6; CI 95%:0–10) and three months after treatment (average 2.9 ± 2.7; CI 95%:0–10) (τ: *p* = 0.006) (Figure 4B).

Overall, 34.8% (15 patients) presented mental clouding. Mental clouding scores showed statistically significant differences between the baseline (average 2.5 ± 3.4; CI 95%:0–8) and three months after treatment (average 1.2 ± 1.9; CI 95%:0-7) (τ: *p* = 0.03) (Figure 4C).

**Figure 5:**
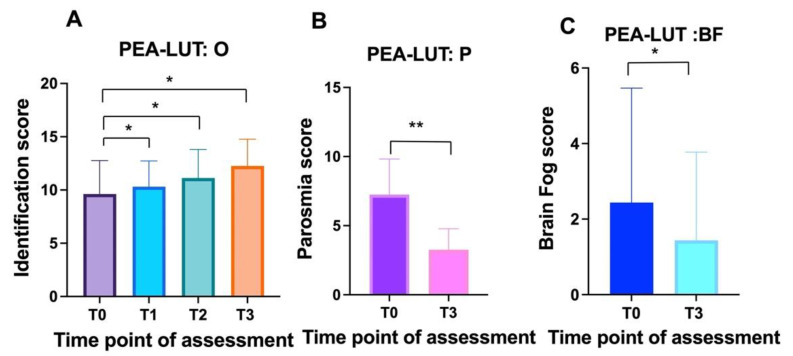
Effect of PEA-LUT treatment without olfactory training on olfaction, parosmia, and mental clouding in patients without prior olfactory training (Naïve 2 group): (**A**) O (olfaction) results of identification test at T0 (baseline), T1 (1 month), T2 (2 months), and T3 (3 months); (**B**) P (parosmia) before (T0) and after three months (T3) of treatment; (**C**) BF (brain fog/mental clouding) before (T0) and after three months (T3) of treatment. **: *p* < 0.01; *: *p* < 0.05.

The Naïve 2 group (no prior history of olfactory training; treated with PEA-LUT alone) included 16 patients (10 men and 6 women) with an average age of 37.6 ± 8.4. These patients had been affected by olfactory function for 8.4 ± 1.7 months on average. We observed an improvement in TDI scores (ANOVA: *p* = 0.03). Statistically significant differences in TDI scores were observed between T0 (average 9.6 ± 3.1; CI 95%: 6–13) and T3 (average 12.2 ± 2.5; CI 95%: 6–14) (Tukey: *p* = 0.02). No differences were observed when comparing T0 and T1 (average 10.3 ± 2.4; CI 95%: 5–12) (*p* = 0.5), T0 and T2 (average 11.1 ± 2.7; CI 95%: 4–13) (*p* = 0.1), T1 and T2 (*p* = 0.8), T1 and T3 (*p* = 0.3), and T2 and T3 (*p* = 0.8) (Figure 5A).

Overall, 93.7% (15 patients) suffered from parosmia. Statistically significant differences were observed in the quality of smell alteration (parosmia) between the baseline (average 7.2 ± 2.6; CI 95%:0–10) and three months after treatment (average 3.2 ± 1.5; CI 95%:0–6) (τ: *p* < 0.00001) (Figure 5B).

Furthermore, 43.7% (7 patients) were affected by mental clouding. No statistically significant differences were observed in mental clouding scores between the baseline (average 2.4 ± 3; CI 95%:0–7) and three months after treatment (average 1.4 ± 2.3; CI 95%: 0–7) (τ: *p* = 0.3) (Figure 5C).

**Figure 6:**
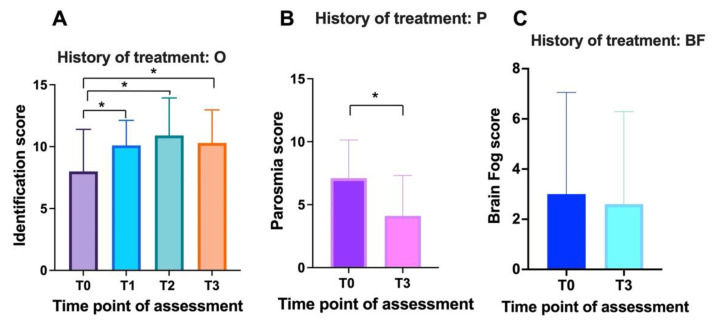
Effect of PEA-LUT treatment plus olfactory training on olfaction, parosmia, and mental clouding in patients with prior olfactory training (previously treated group): (**A**) O (olfaction) results of identification test at T0 (baseline), T1 (1 month), T2 (2 months), and T3 (3 months); (**B**) P (parosmia) before (T0) and after three months (T3) of treatment; (**C**) BF (brain fog/mental clouding) before (T0) and after three months (T3) of treatment. *: *p* < 0.05.

The group with previous olfactory training comprised 10 patients (8 women and 2 men) with an average age of 47.2 ± 9.9. These patients were affected by smell alteration for an average of 8.8 ± 2.6 months. We observed an improvement in TDI scores over the course of the study (ANOVA: *p* = 0.03). Differences in TDI scores were observed between T0 (average 8 ± 3.9; CI 95%: 3–12) and T3 (average 10.9 ± 3; CI 95%: 8–14) (Tukey: *p* = 0.02). No differences were observed when comparing T0 and T1 (average 10.1 ± 2; CI 95%: 7–12) (*p* = 0.5), T0 and T2 (average 10.3 ± 2.6; CI 95%: 6–13) (*p* = 0.1), T1 and T2 (*p* = 0.8), T1 and T3 (*p* = 0.3), and T2 and T3 (*p* = 0.8) (Figure 6A). In this group, 90% (9 patients) presented parosmia. Statistically significant differences were observed in the quality of smell alteration (parosmia) between the baseline (average 7.1 ± 3; CI 95%: 0–10) and three months after treatment (average 4.1 ± 3.2; CI 95%:0–9) (τ: *p* = 0.04) (Figure 6B). Only 40% (4 patients) in this group suffered from mental clouding. No differences were observed in mental clouding scores between the baseline (average 3 ± 4; CI 95%: 0–10) and three months after treatment (average 2.6 ± 3.7; CI 95%: 0–10) (τ: *p* = 0.8) (Figure 6C).

### 3.3. Between-Group Comparison

#### 3.3.1. Smell Quantity

Statistically significant differences were observed at T0 between the Naïve and PEA-LUT and previously treated groups (*p* = 0.04) (Cohen δ = 0.7). No statistically significant differences were observed at T0 between the PEA-LUT and previously treated groups. No statistically significant differences were observed between the three groups at T1, T2, and T3.

#### 3.3.2. Smell Quality (Parosmia)

No differences in the prevalence of parosmia were observed between the three groups over time.

#### 3.3.3. Memory

No differences in the prevalence of mental clouding were observed between the three groups at baseline, but significant differences were observed between the Naïve, PEA-LUT, and previously treated groups (*p* = 0.03) at T3 (Cohen δ = 0.4). No differences were observed between the three groups at T0 or at T3 (*p* > 0.05).

## 4. Discussion

We observed improvements in olfaction, parosmia, and mental clouding among individuals receiving daily ultra-micronized PEA-LUT (700 + 70), with the most favorable results when combining PEA-LUT and olfactory training. Patients who used PEA-LUT alone demonstrated recovery in TDI and parosmia at T3 but did not achieve a significant improvement in their mental clouding. Similar results were observed in patients who had been previously treated with olfactory training alone. Patients treated with PEA-LUT demonstrated evidence of recovery after one month of using the supplement, earlier than in our multicenter clinical trial, where patients treated with PEA-LUT exhibited a trend toward improvement at one month and significant olfactory recovery at two months [2].

Our assessment of parosmia, in which we used an assessment tool adapted from prior work on hyperosmia [13], identified distortion of smell in 79.7% of the patients. Although the PEA-LUT regimen was associated with an improvement in parosmia, an incomplete resolution was common. Parosmia can arise from both central and peripheral damage to the olfactory pathways [14], and persistent smell alteration may reflect perturbations in acetylcholine-mediated signal transmission [15]. We are currently conducting a pilot study testing a precursor of acetylcholine for patients with persistent parosmia after the use of PEA-LUT.

Mental clouding was less commonly identified than parosmia in this cohort, with an overall prevalence of under 40%; this lower prevalence may reflect the use of stringent inclusion criteria. The PEA-LUT regimen three (3 months treatment) was associated with a reduction in mental clouding, consistent with prior work [9]. As mental clouding in patients with COVID-19 is attributed to neuroinflammation [16], the salutary effects of PEA-LUT likely stem from reduced central neuroinflammation. PEA-LUT has shown promise both in mild cognitive impairment (MCI) [9] and the early phase of Alzheimer’s disease (AD) [17], so its use for mental clouding in post-COVID should be considered in combination with olfactory training. Odors can stimulate regeneration and recovery from smell loss [18].

There were several pertinent negatives in our between-group comparisons. For example, we observed no differences between the groups’ smell recovery at T1, T2, and T3, suggesting that PEA-LUT therapy accounts for the preponderance of benefits even in combined therapy with olfactory training [19]. The prevalence of parosmia was also similar between groups, with no statistically significant differences observed between the groups at the baseline and experimental endpoints, again lending credence to the notion that PEA-LUT, rather than the olfactory rehab, may account for improvement [14]. These data support the hypothesis that neuroinflammation contributes to parosmia and delayed recovery.

Finally, the prevalence of mental clouding was similar among the groups, and the analysis of the memory functions showed that patients who received combined PEA-LUT and olfactory training had an improved resolution of mental clouding relative to individuals who used only the supplement. This observation suggests a link between olfactory function and memory [20]. Thus, combining olfactory rehab and anti-neuroinflammatory therapeutics may benefit patients who suffer from both olfactory and memory dysfunction [21].

### 4.1. Benefits of Ultra-Micronization and Mechanism of Action of PEA-LUT

PEA and LUT have potent neuroprotective and anti-inflammatory properties, and their bioavailability is significantly improved when administered in a micronized versus non-micronized form [22]. Ultra-micronization reduces particle size, improving absorption, metabolism, and therapeutic efficacy, as evidenced by several clinical studies [2,11,12,22]. PEA-LUT microgranules can be sublingually administered, which further enhances efficacy by minimizing the loss of activity from gastrointestinal degradation [23]. Therefore, the cellular response to PEA-LUT therapy reflects both the bioavailability and biological activity of the molecules within the central nervous system. 

PEA exerts its effects by modulating histamine release and microglial state. PEA reduces mast cell degranulation (through the autacoid local injury antagonism mechanism [24]). PEA also confers neuroprotection in the CNS by shifting microglia from the M1 pro-inflammatory phenotype to M2 anti-inflammatory phenotype [25]. M2 microglia attenuate neuroinflammation and improve remyelination, promoting the recovery of olfactory pathways and memory [9]. These functions are clinically [9] and anatomically [26] interrelated, as evident in several neurodegenerative disorders affecting smell and memory. PEA also blocks peripheral mast cell activation and signaling pathways from the periphery to the brain; mast-cell–microglia crosstalk plays a pivotal role in neuroinflammation [27].

Two related cellular and molecular mechanisms have been implicated in PEA’s effects: PEA directly activates the nuclear receptor peroxisome proliferator-activated receptor α (PPAR-α), which regulates genic expression [28]. Its action on PPAR-α is responsible for an increased expression of the type 2 cannabinoid receptor (CB2) and the activation of transient receptor potential vanilloid 1 (TRPV1). Moreover, PEA acts through an entourage effect, increasing the availability of endocannabinoid anandamide (AEA) and 2-arachydonoylglycerol (2-AG), which, in turn, directly interacts with CB2, TRPV1, and cannabinoid receptor type 1 (CB1) [24]. Both mechanisms can contribute to olfactory and memory effects.

Luteolin, a flavonoid with anti-inflammatory and neuroprotective properties, exerts its effects through neurotrophic and antioxidant mechanisms. Luteolin increases the expression of the brain-derived neurotrophic factor (BDNF), which supports neuronal survival, growth, and plasticity; BDNF is essential for learning and memory. Luteolin also promotes the antioxidant response by reducing intracellular reactive oxygen species (ROS) in neurons [29]. The reduction in circulating ROS diminishes the overall inflammatory milieu and reduces the proportion of M1 pro-inflammatory microglial cells [30]. The decreased ROS also improves mitochondrial function, ameliorating the brain environment and facilitating the recovery of neuronal connections [30]. In preclinical studies of neuroinflammatory disorders, co-ultra-micronized PEA and luteolin improve neuroprotection compared with either molecule alone [31,32,33].

### 4.2. Study Limitations

This study has several limitations, including its limited sample size, the different sizes of the three groups, and the absence of an untreated control group, given the ethical concerns imperative to offer therapeutic options. Using Cohen’s δ test, we identified the potential for different group sizes to affect our analysis, and larger studies with balanced groups are necessary to corroborate our results. Secondly, given the modest sample size, our results should be considered preliminary. Moreover, we did not consider co-factors (e.g., smoking history), which might adversely affect the health and function of the nasal mucosa and neuroepithelium. Third, we used a previously published method for measuring mental clouding and parosmia, but this tool is not well-validated due to the limited published experience with COVID-19 brain fog/ mental clouding, and it relies on subjective patient assessments.

Additionally, although we used an already validated questionnaire to evaluate the presence of mental clouding/brain fog, we asked our patients to self-report their discomfort and this, because of the different factors influencing the self-perception of the disease, could impact the results. Future research efforts may involve more rigorous testing, assessing criterion, content, and construct validity along with an assessment of reliability and risk of bias. Lastly, olfactory scoring relied on odor identification, and the lack of a comprehensive battery of olfactory assessments incorporating thresholds and detections may have affected results.

## 5. Conclusions

This longitudinal study supports the efficacy of PEA-LUT combined with olfactory training in treating chronic olfactory dysfunction after COVID-19 infection and mental clouding. The combination of PEA-LUT and olfactory rehab had a positive influence on memory independent of the treatment used before enrollment in the study. The putative benefit of PEA-LUT on parosmia requires further study, including biomarkers of inflammation and longer-term follow-up, given the lack of rigorously validated methods and debilitating effects of parosmia on the quality of life for affected individuals.

## Data Availability

Data are available under request to the corresponding author.
